# Alcohol's harm to others in 2021: Who bears the burden?

**DOI:** 10.1111/add.16205

**Published:** 2023-04-25

**Authors:** Anne‐Marie Laslett, Robin Room, Sandra Kuntsche, Dan Anderson‐Luxford, Bree Willoughby, Christopher Doran, Rebecca Jenkinson, Koen Smit, Diana Egerton‐Warburton, Heng Jiang

**Affiliations:** ^1^ Centre for Alcohol Policy Research La Trobe University Melbourne Australia; ^2^ National Drug Research Institute Curtin University Perth Australia; ^3^ Melbourne School of Global and Population Health University of Melbourne Melbourne Australia; ^4^ Social Research Centre on Alcohol and Drugs, Department of Public Health Sciences Stockholm University Stockholm Sweden; ^5^ Cluster for Resilience and Wellbeing, Manna Institute Central Queensland University Brisbane Australia; ^6^ Australian Institute of Family Studies Melbourne Australia; ^7^ Burnet Institute Melbourne Australia; ^8^ School of Clinical Sciences at Monash Health Monash University Melbourne Australia; ^9^ Monash Health Melbourne Australia

**Keywords:** Alcohol, family and other relationship effects, harm to others, heavy episodic drinking, household, inequalities, survey

## Abstract

**Background and aims:**

Alcohol's harm to others (AHTO) has become a key driver of national and international alcohol policy. This study aimed to produce a contemporary, comprehensive estimate of the correlates and harms from others' drinking in 2021 in Australia.

**Design, setting, participants and measurements:**

Across Australia, 2574 adults (1380 women; 1172 men) were sampled via two cross‐sectional survey modes: a random‐digit dial mobile phone sample of 1000 people and 1574 people from the Life in Australia™ panel survey. In 2021 participants were asked about harms they had experienced from the drinking of family, friends, co‐workers and the public in the past year. Applying combined sample weights from each mode, bivariable and adjusted multivariable logistic regressions were used to analyse differences in rates of AHTO by participant gender, age, residence in rural or metropolitan regions, country of birth, education and employment.

**Findings:**

In 2021, 23.6% reported being negatively affected by strangers' drinking and 21.3% by the drinking of someone they knew, with 34.3% reporting being negatively affected a lot or a little by either; 42.4% of respondents reported specific harms from strangers' drinking. Thus, 48.1% of respondents reported any harm (negative effects or specific harms) from others' drinking. Women, younger people, Australian‐born and heavier episodic drinkers reported significantly higher rates of AHTO compared with other respondents. Smaller percentages (7.5%) of participants reported being harmed substantially by others' drinking, including by people they knew (5.8%) or strangers (2.3%). Stratified analyses showed that heavier drinking, furloughed, younger men who were born overseas in English‐speaking countries were affected by others' drinking, whereas women were affected regardless of these factors (apart from age).

**Conclusions:**

More than one‐third of Australian adults appear to have been negatively affected by others' drinking in 2021, with women, younger people and heavier drinkers at greater risk. Substantial harm appears to be more likely to arise from the drinking of people Australians know than from strangers' drinking.

## INTRODUCTION

Alcohol consumption is one of the world's leading avoidable factors for death and disease [1]. However, alcohol not only negatively impacts people who drink; it also affects those close to the drinker (family, friends, work colleagues), as well as society more broadly [2]. Alcohol's harm to others (AHTO) occurs in the home, in licensed premises and on the streets—in almost any public or private place. Along with harms to community amenity such as litter or noise, AHTO includes serious harms to specific others such as violence, abuse, threats, property damage or assault [3, 4]. More than half a million alcohol‐related presentations to emergency departments in Australia occur each year, and it is estimated that 22% of this harm results from AHTO [5]. The estimated annual cost of alcohol to those around the drinker and to agencies responding to AHTO is substantial; for Australia, it was recently estimated to be approximately AU$19.8 billion in 2016—on the same scale as the costs estimated (AU$18.1 billion) to the drinker and for care of drinkers [6]. However, research, treatment and policy still focus mainly on harms to drinkers themselves, rather than also including AHTO [7, 8].

Rates of several key indicators of AHTO in Australia, such as rates of family violence [9, 10] and child protection cases [11], are increasing, but only a handful of items and no comprehensive national survey data on AHTO are collected in a regular, ongoing manner [12]. There is a need to understand the magnitude of and potential increases in costs of AHTO in the wider Australian society, and whether and where these are inequitably distributed in the population [13]. While it is well‐recognized that socio‐economically disadvantaged versus advantaged drinkers suffer more harm per litre [14, 15] this has not been well studied in Australia, and there have been limited systematic studies of socio‐economic differences in AHTO in Australia since 2008 [3]. Isolation also plays a role wherein rural Australians drink more per capita than metropolitan residents [16], and this may result in differences in the risk of experiencing AHTO.

A comprehensive estimate of alcohol's harm to others has not been undertaken for 15 years. Since 2008, the population survey methods landscape has altered [17], there have been significant declines in drinking patterns, particularly among young people [18], and in 2020–21 COVID‐19 led to widespread changes in the ways in which people around the world drank, including in Australia [19, 20]. The first aim of the present study is to produce a comprehensive contemporary survey estimate of the harm arising from others' drinking in Australia. Secondly, we aim to study the disparities in AHTO, including assessment of variations in the experience and level of harm by gender, age, socio‐economic differences and levels of consumption.

## METHODS

### AHTO survey data

Widespread and dramatic decreases in response rates to older land‐line and mobile phone survey methods are forcing changes in population survey methodology [17]. In Australia, the majority of the population refuses to participate in telephone surveys [21]. Concerns about random‐digit dialling (RDD) response rates and the expense of conducting computer‐assisted telephone surveys in Australia led us to split our sample and use both RDD telephone and panel survey techniques. Despite the decline in response rates, there is some evidence that with appropriate fieldwork and analytical techniques there is not necessarily an increase in non‐response bias, and some surveys with poor response rates also find similar results to surveys with better response rates [22].

National cross‐sectional data were collected using two methods: survey component 1, comprising an RDD sample of Australian mobile phone numbers (*n* = 1000); and survey component 2, a sample drawn from the Life in Australia™ (LinA) survey panel [23] (*n* = 1574). The LinA Survey is a probability‐based panel recruited using dual‐frame (land‐line and mobile phone) and RDD that has continued to recruit participants over time using multiple methods, each time ensuring that the data are weighted to be representative of the Australian population [23]. The total *n* (minimum 2500) was determined via power calculations, and the combined sample delivered this number within the project budget. Combining these two components in a data set weighted to be representative, we analysed self‐reported data from 2574 participants aged 18 years and older on the harms they had experienced in the past 12 months from the drinking of families, friends, co‐workers and strangers. The survey was carried out from 3 November to 4 December, 2021. The survey instrument was designed by the authors based on our earlier 2008 survey [3, 24], with input from our partner investigators (see Acknowledgements section), and conducted by a specialist market research and data collection agency [the Social Research Centre (SRC)]. The average time to complete the random‐digit dial survey was just over 30 minutes. The analysis was not pre‐registered, and results should be considered exploratory. The survey was approved by La Trobe University's Human Ethics Research Committee (HEC20518). The study followed the Strengthening the Reporting of Observational Studies in Epidemiology (STROBE) guidelines [25].

### Outcome measures

The survey focused upon participants' experience in the previous 12 months of harms from others' drinking, as well as the participant's own drinking behaviour. Participants were asked questions regarding the extent of harm they experienced because of the drinking of people they knew. First, they were asked whether there were any spouses/partners, housemates, other family members, friends, co‐workers or people in their lives they would consider to be heavy drinkers or people who sometimes drank a lot (hereafter heavy drinkers). A follow‐up question determined whether the heavy drinkers in each relationship category negatively affected the participant in any way over the last 12 months. Next, they were asked about the extent of harm (a little or a lot) they had experienced from the drinking of all the people they knew who had affected them. Respondents negatively affected by the drinking of someone they knew were asked about 21 individual harms (e.g. from being called names to be being physically hurt).

Harm from the drinking of ‘strangers or people you don't know very well’ was then asked separately using 11 items (e.g. been verbally abused, kept awake at night or disturbed). A summary question was asked concerning the extent of negative effect of drinking of strangers (a lot, a little or not at all).

### Socio‐demographic measures

We recorded participants' gender, age, geographical area of residence (metropolitan/regional), education, employment and country of birth. See Table 1 for summary categories.

**TABLE 1:** Sample characteristics and key prevalence estimates of alcohol's harm to others, including negative effects and specific itemised harms^a^, numbers and weighted percentages, Australian Alcohol's Harm to Others Survey, 2021.

Variable	*n* = 2574	Weighted %	CI
Gender			
Men	1172	49.1	(46.5, 51.6)
Women	1380	51.0	(48.4, 53.5)
Age (years)			
18–34	536	30.7	(28.2, 33.3)
35–64	1309	48.4	(45.9, 50.9)
65+	727	21.0	(19.2, 22.9)
Region			
Capital city	1681	66.7	(64.4, 69.0)
Rest of state	860	33.3	(31.0, 35.7)
Employment			
Currently employed	1483	61.6	(59.2, 64.0)
Furloughed^a^	107	3.9	(3.1, 5.0)
Not working	971	34.4	(32.1, 36.8)
Country of birth			
Australia	1818	66.2	(63.7, 68.7)
NESB	406	23.1	(20.8, 25.5)
Mainly ESB	343	10.7	(9.4, 12.2)
Education^a^			
Less than high school	205	9.5	(8.1, 11.0)
Completed high school	1118	62.8	(60.5, 65.1)
University graduate	1235	27.7	(25.7, 29.8)
Participant drinking of 5+ standard drinks in the one occasion			
Not in last year or ever	1467	56.0	(53.5, 58.5)
Less than weekly (monthly or less)	822	33.0	(30.7, 35.4)
1 or 2 days/week	163	6.7	(5.5, 8.0)
3+ days/week	109	4.4	(3.5, 5.5)
Presence of drinker and adverse effects of others' drinking			
Have a heavy or episodic drinker in life	1585	60.2	(57.7, 62.7)
Affected a lot by the drinking of people you know	159	5.8	(4.7, 7.1)
Affected a little by the drinking of people you know	400	15.5	(13.8, 17.4)
Reported negative effects of the drinking of people you know	559	21.8	(19.8, 23.9)
Affected a lot by strangers' drinking	51	2.3	(1.6, 3.1)
Affected a little by strangers' drinking	539	21.3	(19.3, 23.4)
Reported negative effects of strangers' drinking	590	23.5	(21.5, 25.7)
Affected a lot by others' drinking	199	7.5	(6.3, 8.9)
Affected a little by others' drinking	689	26.8	(24.6, 29.0)
Reported negative effects of anyone's drinking	888	34.3	(31.9, 36.7)
Adding specific items			
Reported any harm, including specific items, from strangers' drinking^a^	1067	42.4	(40.0, 44.9)
Reported any harm from others' (known persons' and strangers') drinking, including specific items	1226	48.1	(45.6, 50.6)

Twenty‐two participants were non‐binary or did not report their gender. Small numbers of participants did not answer socio‐demographic, drinking and harm questions and consequently numbers do not total to 2574 participants. ESB = English‐speaking background country of birth; NESB = non‐English‐speaking country of birth; CI = confidence interval.

^a^ Specific harms included verbally abused; physically abused; threatened; involved in a serious argument; forced or pressured into sexual activity; involved in a traffic accident; had property damaged (e.g. house or car); had personal belongings damaged (e.g. clothes); kept awake at night or disturbed; annoyed by people vomiting, urinating or littering; and felt unsafe in a public place.

### Drinking variables

Heavy episodic drinking (HED; drinking five or more drinks on one occasion) was reported as not in the previous 12 months or ever; less than weekly (i.e. including monthly); 1 or 2 days a week; or 3 or more days a week.

### Analysis

RDD and panel subsample differences were evaluated regarding their prediction of key rates and relationships. No significant differences were found, justifying combination. Weights were created for each sample separately to increase representativeness, and these formed the basis for a combined overall weight (see Supporting information, Table S1). A small percentage (88, 7.6%) of 1154 participants discontinued the interview, yielding 1066 completions in our valid RDD sample. These 88 uncompleted questionnaires were coded as invalid and excluded from the weighting approach and regression analyses.

In all analyses, participant numbers, weighted prevalence, weighted effect estimates and confidence intervals (CIs) are presented. Patterns of variations in AHTO were examined for different relationship types (spouse/partner, housemate, other family member, friend, co‐worker and stranger). Non‐overlapping CIs provide a conservative indication of statistically significant differences in estimated percentages of outcomes, with key differences tested further. Weighted bivariable (unadjusted) and multivariable logistic regression analyses were used to measure how the prevalence (any versus no) and level (a lot versus a little or none) of AHTO differed by gender, age, rurality and measures of socio‐economic differences, including education and employment (separately and then adjusting for all these variables in the model, unless otherwise specified). Education and employment were highly correlated with each other and, while both were significantly associated with AHTO at the bivariable level, only employment was added into the multivariable model. The relationship of the reporting of AHTO with the participant's own drinking pattern in terms of heavy episodic drinking was also examined. A stratified analysis was conducted to study the risks of reporting alcohol‐related harms from others' drinking for women and men.

### Response rate

The response rate for the RDD survey was 5.5%. For the in‐scope population for the survey selected from active LinA panel members, the cooperation rate (the percentage of interviews/interviews + refusals) was 18.1%. For the panel survey, a total of 2003 active panel members were invited to take part, and 1574 (78.6%) completed the survey. The panel's cumulative response rate, taking account of the recruitment rate into the panel and the retention rate, was 6.1% [23]. Low response rates are currently common in Australia and many high‐income countries [21], yet despite concerns the weighted results have been found to be representative, as is considered further in the Discussion section. The de‐identified data that support the findings of this study are available on request from the corresponding author. The data are not publicly available due to privacy or ethical restrictions.

## RESULTS

Characteristics of the sample and key prevalence estimates are reported in Table 1. Similar percentages of Australians reported that they had been negatively affected (either a lot or a little) from the drinking of people they did not know (strangers, 23.6%, CI = 19.5, 28.2) and people they knew (21.8%, CI = 19.8%, 23.9%). Substantial harm was reported by 7.5% of participants who reported being harmed ‘a lot’ by others' drinking (CI = 6.3%, 8.9%), with 5.8% (CI = 4.7%, 7.1%) reporting being harmed a lot by a known drinker, significantly more than the 2.3% (CI = 1.6%, 3.1%) who reported being harmed a lot by a stranger's drinking. Conversely, respondents reported a statistically significantly higher prevalence of being negatively affected a little by strangers' drinking than by known drinkers (21.3 versus 15.5%). Positive responses to one or more of these specific questions regarding harms from strangers' drinking were given by 42.4% of respondents. Combining this measure of specific harms with the 21.8% negatively affected by known drinkers, almost half (48.1%) of respondents gave some indication of having experienced any harmful effects from drinking by others during the previous 12 months.

Table 2 provides prevalence estimates and CIs on AHTO from people in various relationships with the participant. Women reported harms from household members' and other family members' drinking more commonly than men. Bivariable logistic regression analyses for the various reports of types of AHTO by relationship confirmed that women were significantly more likely than men to report harm from people with whom they lived [odds ratio (OR) = 2.0, CI = 1.2, 3.2] and from relatives and intimates with whom they did not live (OR = 2.0, CI = 1.4, 2.8), with these differences driving greater experience of harm by women than men from all categories of drinkers people knew (OR = 2.1, CI = 1.5, 2.8). No significant differences were found between men and women in harm from others' drinking in the friendship and co‐worker categories. Women reported slightly more harm from strangers' drinking than men (OR = 1.3, CI = 1.0, 1.6). Moreover, women reported more harms than men from others' drinking across all categories of relationship including strangers (OR = 1.3, CI = 1.1, 1.6).

**TABLE 2:** Alcohol's harm to others by different categories of relationship between the participant and the person whose drinking was held responsible for harm, raw numbers, weighted percentages and confidence intervals, by gender and age group, Australian Alcohol's Harm to Others Survey, 2021.

	Women			Men			Total		
	18–34	35–64	65+	Subtotal	18–34	35–64	65+	Subtotal	
*n*	283	722	373	1380*	240	578	354	1172*	2574
Negative effects from a known person^a^									
Household member	5.4 (3.2, 8.9)	7.7 (5.3, 10.9)	3.7 (1.9, 7.0)	6.1 (4.7, 8.1)** (*P* = 0.000)	1.0 (0.0, 2.5)	4.5 (2.8, 7.3)	3.6 (1.6, 7.5)	3.2 (2.2, 4.8)	4.7 (3.8, 5.9)
Relative or intimate (not in household)	13.6 (9.818.6)	15.2 (12.1, 19.0)	10.7 (7.3, 15.4)	13.8 (11.7, 16.3)*** (*P* = 0.000)	6.8 (3.8, 11.9)	8.9 (6.4, 12.1)	5.3 (3.2, 8.8)	7.5 (5.8, 9.6)	10.7 (9.3, 12.3)
Household member, relative or intimate (pooled)	17.6 (13.2, 23.0)	21.6 (17.8, 25.9)	13.1 (9.4, 18.0)	18.6 (16.1, 21.4)*** (*P* = 0.000)	7.7 (4.5, 12.8)	12.1 (9.2, 15.9)	8.3 (5.3, 12.6)	10.0 (8.0, 12.4)	14.4 (12.7, 16.2)
Friend	7.9 (4.8, 12.7)	6.2 (4.5, 8.6)	4.2 (2.2, 7.8)	6.3 (4.9, 8.2) (*P* = 0.2229)	6.7 (3.9, 11.3)	10.9 (8.0, 14.7)	3.2 (1.7, 5.8)	7.9 (6.2, 10.2)	7.1 (5.9, 8.5)
Co‐worker	2.0 (0.9, 4.4)	4.2 (2.6, 6.7)	*n* < 5	2.7 (1.8, 4.0) (*P* = 0.5907)	3.1 (1.2, 7.5)	4.6 (2.8, 7.3)	0.1 (0.0, 0.4)	3.1 (2.0, 4.8)	2.9 (2.1, 3.9)
Friend, co‐worker, other (pooled)	9.1 (5.8, 14.0)	9.6 (7.2, 12.6)	4.2 (2.2, 7.8)	8.3 (6.6, 10.4) (*P* = 0.4044)	8.8% (5.4, 14.0)	12.9, (9.8, 16.9)	3.2 (1.8, 5.8)	9.6 (7.7, 12.0)	8.9 (7.6, 10.5)
Any of the above known people									
Household member, relative, intimate, friend, co‐worker, other (pooled)	26.3 (20.7, 32.7)	26.7 (22.6, 31.1)	16.3 (12.0, 21.7)	24.4 (21.6, 27.5)* (*P* = 0.0116)	16.2 (11.3, 22.7)	23.6 (19.5, 28.2)	12.9 (9.1, 17.9)	19.1 (16.3, 22.1)	21.8 (19.8, 23.9)
AHTO‐specific^b^ harms to strangers	58.8 (51.7, 65.7)	46.4 (41.7, 51.2)	20.3 (15.5, 26.3)	45.1 (41.7, 48.6)* (*P* = 0.0232)	46.0 (38.5, 53.6)	41.7 (36.7, 46.8)	25.5 (20.3, 31.6)	39.3 (35.8, 42.9)	42.4 (40.0, 44.9)
Any AHTO—all relationship types and strangers (pooled)	62.7 (55.5, 69.3)	52.8 (48.1, 57.5)	30.1 (24.2, 36.7)	51.3 (47.9, 54.8)** (*P* = 0.0079)	49.4 (41.8, 57.1)	48.3 (43.2, 53.4)	29.7 (24.2, 36.0)	44.5 (40.9, 48.2)	48.1 (45.6, 50.6)

AHTO = alcohol's harm to others. Weighted percentages and confidence intervals are presented. Bivariable logistic regression analyses were undertaken to assess significance of gender differences at the total level (male versus/female subtotal), but the odds ratios are not presented in the table; only statistical significance of these analyses are using standard P‐value nomenclature:

*** P < 0.001,

** P < 0.01 and

* P < 0.05.

^a^ The 21 harms asked about the drinking of people known to the respondent included being negatively affected at a social occasion, emotionally hurt or neglected, having a serious argument that did not include physical violence, failing to do something they were being counted on to do, having to take a person somewhere or pick them up, spending time caring for them, having to stop seeing them, cleaning up after them, being threatened by them, taking on extra caring responsibilities for children or others, something being broken or damaged which mattered to the respondent, house or property damage, the drinker not doing their share of work around the house, having financial trouble, being put at risk in the car when the drinker was driving, not being able to bring friends home, being physically hurt by the drinker, having to leave home to stay somewhere else, being forced or pressured into sex or something sexual, having family problems or being called names or otherwise insulted.

^b^ Specific harms from strangers' drinking included: verbally abused; physically abused; threatened; involved in a serious argument; forced or pressured into sexual activity; involved in a traffic accident; had property damaged (e.g. house or car); had personal belongings damaged (e.g. clothes); kept awake at night or disturbed; annoyed by people vomiting, urinating or littering; and felt unsafe in a public place.

Table 2 also provides information on age differences in AHTO. The younger and middle‐aged groups generally reported more harm than the oldest age group for all relationship categories. We extended this analysis by comparing the oldest and the youngest groups, combining men and women, finding significantly decreased odds of harm from AHTO in the oldest versus youngest age group for harms from the drinking of co‐workers (OR = 0.01, CI = 0.00, 0.10), friends (OR = 0.48, CI = 0.26, 0.87) and strangers (OR = 0.27, CI = 0.20, 0.36). The odds of the oldest group experiencing any AHTO overall were 68% lower than in the youngest group (OR = 0.32, CI = 0.24, 0.43).

Table 3 shows that being female (versus male) and being younger (versus older) was associated with more experience of any AHTO in both the bivariable and multivariable models. Not working was associated with less experience of AHTO in the bivariable logistic regression, although this association was not significant in the multivariable logistic regression. Employment and education were significantly associated with AHTO at the bivariable level and employment was included but not significant in the multivariable model. In both models, compared to abstainers and non‐risky drinkers, participants who drank less than weekly (monthly or several times a month) in a heavy episodic way were more likely to report AHTO. In the adjusted model, respondents who reported HED three or more times a week were also more likely to report any AHTO than abstainers.

**TABLE 3:** Relative odds of reporting any harm from the drinking of known drinkers or strangers, by participant demographics, Australian Alcohol's Harm to Others Survey, 2021: bivariable and multivariable logistic regression.

		Bivariable logistic regression				Multivariable logistic regression^a^			
	*n* = 2574	Unadjusted odds ratio	95% CI		*P*‐value	Adjusted odds ratio	95% CI		*P*‐value
Gender									
Men	1172	1.00				1.00			
Women	1380	1.32**	1.08	1.61	0.008	1.36**	1.09	1.69	0.007
Age (years)									
18–34	536	1.00				1.00			
35–64	1309	0.79	0.61	1.01	0.062	0.74*	0.57	0.97	0.026
65+	727	0.33***	0.24	0.44	0.000	0.33***	0.23	0.47	0.000
Region									
Capital city	1681	1.00				1.00			
Rest of state	860	1.05	0.85	1.30	0.624	1.06	0.85	1.33	0.598
Employment									
Currently employed	1483	1.00				1.00			
Furloughed^b^	107	1.55	0.92	2.60	0.101	1.59	0.91	3.13	0.106
Not working	971	0.60***	0.49	0.75	0.000	0.94	0.71	1.23	0.629
Country of birth									
Australia	1818	1.00				1.00			
NESB	406	0.78	0.59	1.02	0.073	0.73*	0.55	0.99	0.040
Mainly ESB	343	1.07	0.80	1.45	0.636	1.32	0.96	1.80	0.087
Participant drinking of 5+ drinks in a session									
Not in last year or ever	1467	1.00				1.00			
Less than weekly (monthly)	822	1.57***	1.26	1.97	0.000	1.35*	1.06	1.72	0.014
1 or 2 days/week	163	1.02	0.68	1.54	0.921	0.93	0.61	1.43	0.749
3+ days/week	109	1.63	1.00	2.66	0.052	1.82*	1.10	3.02	0.021
Education^a^									
Less than high school	205	1.00							
Completed high school	1118	1.46*							
University graduate	1235	1.47*							

Number used in multivariable logistic regression: *n* = 2492. ESB = English‐speaking background country of birth; NESB = non‐English‐speaking country of birth; CI = confidence interval.

^a^ Model adjusted for gender, age, region, employment and country of birth; education not included in adjusted multivariable model as education and employment were highly correlated.

^b^ Furloughed: temporarily laid off during COVID.

* *P* < 0.05;

** *P* < 0.01;

*** *P* < 0.001.

Table 4 presents information on the bivariable and adjusted multivariable logistic regression results obtained for participants who reported being harmed a lot versus a little or not at all. Women, rural and regional residents, those who were temporarily not working (furloughed) and those who reported HED at least 3 days a week were significantly more likely to report being harmed substantially in both the bivariable and multivariable models. Younger people were more likely to report being harmed substantially only at the multivariable level. Education was not significant at the bivariable level and was not included in the overall multivariable model.

**TABLE 4:** Weighted unadjusted and adjusted odds of reporting being negatively affected substantially (a lot versus not at all or a little) from others' (known or stranger) drinking, Australian Alcohol's Harm to Others Survey, 2021.

		Bivariable logistic regression				Multivariable logistic regression^a^			
	*n* = 2574	Unadjusted odds ratio	95% CI		*P*‐value	Adjusted odds ratio	95% CI		*P*‐value
Gender									
Men	1172	1.00				1.00			
Women	1380	2.00**	1.35	2.97	0.001	1.94**	1.27	2.96	0.002
Age (years)									
18–34	536	1.00				1.00			
35–64	1309	1.22	0.78	1.92	0.387	1.08	0.67	1.75	0.735
65+	727	0.62	0.36	1.09	0.096	0.47*	0.25	0.88	0.018
Region									
Capital city	1681	1.00				1.00			
Rest of state	860	1.74**	1.19	2.55	0.004	1.66*	1.11	2.48	0.013
Employment									
Currently employed	1483	1.00				1.00			
Furloughed^b^	107	2.79**	1.33	5.85	0.007	2.70*	1.20	6.07	0.017
Not working	971	0.96	0.65	1.43	0.852	1.13	0.69	1.86	0.625
Country of birth									
Australia	1818	1.00				1.00			
Mainly NESB	406	0.69	0.39	1.23	0.211	0.78	0.44	1.37	0.382
Mainly ESB	343	0.99	0.56	1.74	0.973	1.09	0.60	1.99	0.767
Participant drinking of 5+ drinks in a session									
Not in last year or ever	1467	1.00				1.00			
Less often (monthly)	822	0.91	0.60	1.36	0.633	0.86	0.56	1.31	0.477
1 or 2 days/week	163	0.62	0.25	1.55	0.308	0.70	0.27	1.82	0.469
3+ days/week	109	2.00*	0.90	4.15	0.093	2.39*	1.06	5.39	0.036
Education^a^									
Less than high school	205	1.00							
Completed high school	1118	1.29	0.65	2.53	0.470				
University graduate	1235	0.97	0.48	1.97	0.929				

Number used in multivariable logistic regression: *n* = 2483. ESB = English‐speaking background country of birth; NESB = non‐English‐speaking country of birth; CI = confidence interval.

^a^ Model adjusted for gender, age, region, employment and country of birth and participant drinking. Education not included in adjusted multivariable model as education and employment were highly correlated.

^b^ Furloughed: temporarily laid off during COVID.

* *P* < 0.05;

** *P* < 0.01.

In Tables 3 and 4, the odds ratios for harm for participants who reported HED three or more times per week were large, indicating that HED was a strong risk factor for suffering alcohol‐related harms from others. Female gender was also strongly associated with alcohol‐related harms to others. Thus, stratified analyses were conducted to analyse risks of being a victim of alcohol‐related harms for women and men and participants who did and did not report HED. Stratifying by gender (see Table 5), among men, the youngest age group (versus the oldest), being furloughed and being born in country where English is primarily spoken (versus being born in Australia) and more frequent HED were significant predictors for experiencing harms from others' drinking. In contrast, only younger age predicted experiencing harm from others' drinking among women. This suggests that women, regardless of varying socio‐demographic backgrounds (aside from age), were likely to report similar levels of harm, yet specific groups of men were at greater risk.

**TABLE 5:** Stratified analyses predicting any harm from the drinking of known drinkers or strangers differ by gender (male versus female), Australian Alcohol's Harm to Others Survey, 2021.

*n* = 2574	Male (*n* = 1172)		Female (*n* = 1380)	
	AOR^a^	*P*‐value	AOR^a^	*P*‐value
Age (years)				
18–34	1.00		1.00	
35–64	0.85 (0.58, 1.25)	0.408	0.65 (0.45, 0.93)*	0.018
65+	0.36 (0.21, 0.59)***	0.000	0.30 (0.18, 0.49)***	0.000
Region				
Capital city	Ref 1.00		1.00	
Rest of state	1.09 (0.79, 1.52)	0.596	1.05 (0.77, 1.42)	0.757
Employment				
Currently employed	1.00		1.00	
Furloughed^b^	2.41 (1.01, 5.79)	0.048	1.27 (0.63, 2.56)	0.505
Not working	1.09 (0.73, 1.63)	0.670	0.82 (0.56, 1.19)	0.293
Country of birth				
Australia	1.00		1.00	
NESB	0.70 (0.47, 1.04)	0.076	0.77 (0.50, 1.19)	0.243
Mainly ESB	1.75 (1.10, 2.77)*	0.018	0.96 (0.63, 1.44)	0.826
Participant drinking of 5+ drinks in a session				
Not in last year or ever	1.00		1.00	
Less than weekly	1.37 (0.97, 1.93)	0.074	1.32 (0.94, 1.85)	0.113
1 or 2 days/week	0.76 (0.45, 1.31)	0.327	1.62 (0.79, 3.32)	0.185
3+ days/week	1.97 (1.10, 3.54)*	0.023	1.12 (0.41, 3.09)	0.826

ESB = English‐speaking background country of birth; NESB = non‐English‐speaking country of birth; AOR = adjusted odds ratio.

^a^ Model adjusted for gender, age, region, employment and country of birth; education not included in adjusted multivariable model as education and employment were highly correlated.

^b^ Furloughed: temporarily laid off during COVID.

* *P* < 0.05;

*** *P* < 0.001.

The predictors of overall harm among respondents who reported HED and non‐HED were quite similar (see Supporting information, Table S1), and age was the only different significant predictor in these two models. One additional statistically significant difference was found in the non‐HED group between the 18–34‐ and 35–49‐year age groups that was not evident in the HED group and the overall group.

## DISCUSSION

Almost half (48.1%) of respondents in this Australia‐wide survey reported experiencing one or more harms from others' drinking in the last year, with 7.5% reporting that they had been negatively affected ‘a lot’ by others' drinking and another 26.8% reporting they had been harmed ‘a little’. Analysing the likelihood of any harm from others' drinking (including specific harms from known drinkers' or strangers' drinking), women, young people, Australian‐born (versus participants born in non‐English‐speaking countries) and occasionally reported HED were more likely to report AHTO.

Women were more likely than men to be negatively affected both by the drinking of people with whom they lived and were related to and by the drinking of strangers. In line with previous research, women and young people were at greater risk of AHTO. More women than men have reported a range of harms from others' drinking, including harms from intimate partners [24] and financial harms [26] from others' drinking. This is consistent with previous findings from many countries, including the previous Australian survey [3, 27, 28]. Young people comprise the group of adults that has consistently reported more harms from strangers' drinking and from drinking of their friends and co‐workers [3, 4, 27, 29]. In our study, harm from strangers' drinking was considerably lower for those aged 65 years or more than for younger participants and significantly higher for women aged 18–34 years than for middle‐aged women. Results in our study are broadly consistent with findings in previous studies, that young people report more harms than older adults from strangers' drinking and from drinking of their friends and co‐workers [3, 4, 27, 29]. This may be because they go out more to drink and socialize and thus are more often in contact with inappropriate behaviour not only from strangers, but also from friends and the people with whom they work [30]. This might also be partly explained by a generational effect, whereby younger generations in higher‐income countries have become more sensitive to violence, harassment and other harms which makes them more likely to report AHTO; alternatively, older people (and other groups) may socialize less in drinking environments and may be more tolerant of or desensitized to harm [31, 32].

People who were abstainers or had not drunk in a heavy episodic manner in the past year were at reduced risk of reporting any AHTO compared with those who reported doing so less than weekly. In comparison to the light and non‐drinkers, participants drinking in this way 3 days or more per week were also more likely to be harmed ‘a lot’ (Table 3). That people who do not drink episodically or at all are less likely to experience AHTO is consistent with previous studies, and results probably from their diminished exposure to a social circle of heavy drinking friends and family members [28]. Abstainers are also less likely to frequent public drinking places where they may interact with strangers who drink heavily, which has been shown to be associated with increased harm [12].

It has been argued that AHTO survey findings focus upon a wide array of harms, but not serious harms [33]. Our results analyse any negative effects from someone's drinking and substantial effects where participants reported that they were harmed ‘a lot’. This study shows that women (versus men), younger (versus older), rural and regional residents (versus capital city), those who were temporarily not working (furloughed) and people who reported HED at least 3 days a week (versus abstainers) were more likely to report substantial harm (compared with no or less harm) after adjusting for all variables in the model. These predictors of substantial harm are similar to those found for predictors of any harm, with some distinctions. For instance, differences were found for rural and furloughed participants wherein these groups were significantly more likely to report substantial harm, although differences were not found for any harm. Conversely, respondents who were in the middle age category and respondents from NESB countries of birth and who reported HED less than weekly were not less likely (as they had been for reporting any harm) to have experienced different rates of substantial harm. It is notable that significant differences by educational level for those affected by any AHTO disappeared for those affected ‘a lot’ by others' drinking.

A limitation of our survey is its low response rate. While there is some evidence that declining response rates do not necessarily cause non‐response bias [22], these results need to be interpreted cautiously, although they are in line with other surveys [3]. We also obtained similar findings from both survey modes when analysed separately, and included the LinA findings as a sensitivity analysis (Supporting information, Table S2). Moreover, this national survey coincided with the COVID‐19 pandemic. In the year prior to the current survey, which was conducted in November–December 2021, COVID‐19 and associated restrictions from February onwards affected the patterns of behaviour and association of many Australians, including their drinking patterns and locations [34]. Alcohol consumption decreased among lighter drinkers but increased among heavier drinkers in Australia [20, 35], and similar patterns were observed in the United Kingdom [36]. In studies in Europe, COVID‐19 was linked to an increase in some drinking‐related harms, including mental ill‐health, family violence and death [37, 38, 39]. Importantly, AHTO might also have been significantly affected by COVID‐19. For instance, lockdown restrictions potentially decreased harm from strangers, meaning that stranger harm in more normal times is likely to be underestimated here, and would likely be even higher if not for COVID‐19.

In 2008, almost three‐quarters of the Australian population reported experiencing at least one of a similar range of harms from others' drinking in the previous 12 months [3]. The prevalence in 2021 was substantially lower, and may be due to changes in response rates [17], significant declines in drinking patterns [40] or COVID‐19‐related changes in drinking and harm [20]. Our findings provide the most up‐to‐date estimates of the burden of AHTO in the general population. Existing studies of risk factors for the global burden of disease [41] have so far only included AHTO in three areas: drink‐driving [42], fetal alcohol syndrome [43] and interpersonal violence [44]. Studies such as the present one underline that standard measures of harm from drinking in the GBD greatly underestimate the burden of harm from drinking and thus alcohol's share in the global burden of disease.

The present findings highlight that in 2021, where behavioural patterns were affected during the preceding year by COVID‐19, women reported substantially more harm than men, particularly from people they knew but also from strangers. This suggests that attention needs to be paid to reducing harm, especially to women (and particularly younger women) from others' drinking. Data focusing upon differences by this and other social inequalities can provide guidance and benchmarking for policy discussions and interventions.

Stratifying our results we found that, among men, middle‐aged and younger men, furloughed men and men who reported HED more often (versus less frequently or not at all) and who were born overseas in countries where English is predominantly spoken (versus born in Australia) were more likely to report harm from others' drinking than their counterparts. In contrast, our results indicate that, among women, more frequent HED by women was not associated with an increased risk of harm from others' drinking. It seems that women report more harm than men and are targeted regardless of how much they drink, whereas harms to men from other's drinking are more associated with their own heavy drinking. Men who more commonly drink and cause more harm than women may end up in heavy drinking situations where aggression and violence towards other men and women occur, with this potentially linked to expectations around men's behaviours and disinhibition in heavy drinking cultures, and more harm to themselves when they report more frequent HED.

The percentages we report, if extrapolated to the Australian population (15+ years) [45], mean that almost 10 million (9.997 million, 48.1%) adults were harmed in any way by another person's drinking during the previous year [45] and 1.56 million were affected substantially. These survey findings complement analyses of response agency data [4, 6] and qualitative data [46] and crucially inform the public, academics and key public policymakers, service providers and decisionmakers about the full externalities of alcohol and the need to take into account alcohol's harm to others in national prevention strategies.

## CONCLUSIONS

Just under half of Australian adults experienced harms from others' drinking in the year prior to the survey, with a third of Australians reporting that this had negatively affected them. Women were more likely to experience these harms. Younger people were at greater risk of experiencing AHTO. People who abstained or always drink fewer than five drinks on an occasion and those born in non‐English‐speaking countries were at reduced risk of reporting AHTO. At the level of substantial harm, higher rates were reported by women, younger, regional/rural and Australian‐born participants and those who themselves frequently drank heavily. For both genders, serious harm is fairly common. An estimated 10 million Australian adults experience harms from other people's drinking each year, with more than 1.5 million experiencing serious harm. The 2021 survey findings will inform policymakers in Australia and elsewhere of the full extent of the harm from others' drinking.

## AUTHOR CONTRIBUTIONS

**Anne‐Marie Laslett:** Conceptualization (lead); formal analysis (equal); funding acquisition (lead); investigation (lead); methodology (lead); project administration (lead); writing—original draft (lead); writing—review and editing (lead). **Robin Room:** Conceptualization (lead); writing—original draft (equal); writing—review and editing (equal). **Sandra Kuntsche:** Conceptualization (lead); formal analysis (supporting); writing—original draft (supporting); writing—review and editing (supporting). **Dan Anderson‐Luxford:** Formal analysis (supporting); project administration (supporting); writing—original draft (supporting); writing—review and editing (supporting). **Bree Willoughby:** Formal analysis (supporting); project administration (supporting); writing—original draft (supporting). **Chris Doran:** Conceptualization (lead); methodology (supporting); writing—original draft (supporting); writing—review and editing (supporting). **Rebecca Jenkinson:** Conceptualization (supporting); writing—review and editing (supporting). **Koen Smit:** Project administration (supporting); writing ‐ original draft (supporting); writing ‐ review and editing (supporting). **Diana Egerton‐Warburton:** Conceptualization (lead); writing—original draft (supporting); writing—review and editing (supporting). **Heng Jiang:** Conceptualization (lead); formal analysis (lead); methodology (equal); writing—original draft (equal); writing—review and editing (equal).

## DECLARATION OF INTERESTS

None to declare.

## Supporting information


**Table S1.** Stratified analyses predicting any harm from the drinking of known drinkers or strangers differ by HED (heavy episodic drinking – 5 + per occasion).
**Table S2.** Relative odds of reporting any harm from the drinking of known drinkers or strangers, by participant demographics, Australian Alcohol's Harm to Others Survey, 2021: multivariable logistic regression using LinA data only.

## Data Availability

The de‐identified data that support the findings of this study are available on request from the corresponding author. The data are not publicly available due to privacy or ethical restrictions.
