# Supplementary material for: Alcohol's harm to others in 2021: Who bears the burden?
Source: Addiction. 2023 Apr 25;118(9):1726–38. doi: 10.1111/add.16205 (PMC10952517; doi:10.1111/add.16205)
Supplement: Supplementary file 1 — Table S1. Stratified analyses predicting any harm from the drinking of known drinkers or strangers differ by HED (heavy episodic drinking – 5 + per occasion). Table S2. Relative odds of reporting any harm from the drinking of known drinkers or strangers, by participant demographics, Australian Alcohol's Harm to Others Survey, 2021: multivariable logistic regression using LinA data only. [file ADD-118-1726-s001.docx]

**Supplementary tables**

**Table S1 Stratified analyses predicting any harm from the drinking of known drinkers or strangers differ by HED (heavy episodic drinking – 5+ per occasion)**

| **(n=2574)** | **HED (N=1107)** |  | **Non-HED (n=1467)** | |
| --- | --- | --- | --- | --- |
|  | **AOR^a^ (95% CI)** | **P-value** | **AOR^a^ (95% CI)** | **P-value** |
| **Gender** |  |  |  |  |
| Men | 1.00 |  | 1.00 |  |
| Women | 1.30 (0.97, 1.73) | 0.079 | 1.39 (1.00, 1.92) | 0.050 |
| **Age** |  |  |  |  |
| 18-34 | 1.00 |  | 1.00 |  |
| 35-64 | 0.94 (0.65, 1.37) | 0.763 | 0.59 (0.41, 0.86)** | 0.006 |
| 65+ | 0.43 (0.27, 0.68)*** | 0.000 | 0.21 (0.41, 0.86)*** | 0.000 |
| **Region** |  |  |  |  |
| Capital city | 1.00 |  | 1.00 |  |
| Rest of state | 1.05 (0.78, 1.42) | 0.749 | 1.12 (0.80, 1.57) | 0.508 |
| **Employment** |  |  |  |  |
| Currently employed | 1.00 |  | 1.00 |  |
| Furloughed ^b^ | 1.64 (0.82, 3.26) | 0.160 | 1.74 (0.67, 4.53) | 0.256 |
| Not working | 1.08 (0.76, 1.51) | 0.674 | 0.81 (0.51, 1.27) | 0.358 |
| **Country of birth** |  |  |  |  |
| Australia | 1.00 |  | 1.00 |  |
| NESB ^c^ | 0.81 (0.56, 1.18) | 0.277 | 0.68 (0.42, 1.10) | 0.116 |
| Mainly ESB ^d^ | 1.31 (0.87, 1.97) | 0.201 | 1.34 (0.81, 2.21) | 0.250 |

*Notes:* ^a^ Model adjusted for gender, age, region, employment and country of birth; education not included in adjusted multivariable model as education and employment were highly correlated; ^b^ Furloughed: temporarily laid off during COVID; ^c^ NESB: non-English speaking country of birth; ^d^ ESB: English-speaking background country of birth; Asterisks indicate statistical significance: *p<0.05, **p<0.01, ***p<0.001.

**Table S2 Relative odds of reporting any harm from the drinking of known drinkers or strangers, by participant demographics, Australian Alcohol’s Harm to Others Survey, 2021: multivariable logistic regression using LinA data only**

|  | **Number**  **(n=1508)** | **Multivariable logistic regression** ^a^ | | | | |  |  |  |
| --- | --- | --- | --- | --- | --- | --- | --- | --- | --- |
|  |  | **Adjusted Odds ratio** | **95% CI** | | | **P-value** | |  |  |
| **Gender** |  |  |  | |  |  | | |  |
| Men | 630 | 1.00 |  | |  |  | | |  |
| Women | 868 | 1.92*** | 1.44 | | 2.55 | 0.000 | | | |
| **Age** |  |  |  | |  |  | | | |
| 18-34 | 248 | 1.00 |  | |  |  | | | |
| 35-64 | 813 | 0.75 | 0.53 | | 1.06 | 0.109 | | | |
| 65+ | 445 | 0.47** | 0.30 | | 0.76 | 0.002 | | | |
| **Region** |  |  |  | |  |  | | | |
| Capital city | 998 | 1.00 |  | |  |  | | | |
| Rest of state | 510 | 1.25 | 0.94 | | 1.67 | 0.119 | | | |
| **Employment** |  |  |  | |  |  | | | |
| Currently employed | 851 | 1.00 |  | |  |  | | | |
| Furloughed ^b^ | 66 | 1.59 | 0.80 | | 3.14 | 0.184 | | | |
| Not working | 582 | 0.81 | 0.57 | | 1.15 | 0.243 | | | |
| **Country of birth** |  |  |  | |  |  | | | |
| Australia | 1068 | 1.00 |  | |  |  | | | |
| NESB ^c^ | 229 | 0.68* | 0.46 | | 0.99 | 0.045 | | | |
| Mainly ESB ^d^ | 208 | 1.28 | 0.85 | | 1.93 | 0.234 | | | |
| **Participant drinking of five plus drinks in a session** |  |  |  |  | |  | | | |
| Not in last year or ever | 859 | 1.00 |  | |  |  | | | |
| Less than weekly | 470 | 1.40* | 1.03 | | 1.91 | 0.032 | | | |
| 1 or 2 days/ week | 97 | 0.95 | 0.56 | | 1.61 | 0.841 | | | |
| 3+ days/week | 71 | 2.05* | 1.08 | | 3.89 | 0.028 | | | |

*Notes:* Number used in multivariable logistic regression: n=2492;^a^ Model adjusted for gender, age, region, employment and country of birth; education not included in adjusted multivariable model as education and employment were highly correlated; ^b^ Furloughed: temporarily laid off during COVID; ^c^ NESB: non-English speaking country of birth; ^d^ ESB: English-speaking background country of birth; Asterisks indicate statistical significance: *p<0.05, **p<0.01, ***p<0.001.
